# MicroRNA Expression in Cutaneous Lupus: A New Window to Understand Its Pathogenesis

**DOI:** 10.1155/2019/5049245

**Published:** 2019-12-30

**Authors:** Silvia Méndez-Flores, Janette Furuzawa-Carballeda, Gabriela Hernández-Molina, Gustavo Ramírez-Martinez, Nora E. Regino-Zamarripa, Blanca Ortiz-Quintero, Luis Jiménez-Alvarez, Alfredo Cruz-Lagunas, Joaquín Zúñiga

**Affiliations:** ^1^Department of Dermatology, Instituto Nacional de Ciencias Médicas y Nutrición Salvador Zubirán, Mexico City, Mexico; ^2^Department of Immunology and Rheumatology, Instituto Nacional de Ciencias Médicas y Nutrición Salvador Zubirán, Mexico City, Mexico; ^3^Laboratory of Immunobiology and Genetics, Instituto Nacional de Enfermedades Respiratorias Ismael Cosío Villegas, Mexico City, Mexico; ^4^Tecnologico de Monterrey, Escuela de Medicina y Ciencias de la Salud, Mexico City, Mexico; ^5^Department of Biochemistry, Instituto Nacional de Enfermedades Respiratorias Ismael Cosío Villegas, Mexico City, Mexico

## Abstract

**Background:**

The role of miRNAs in the pathogenesis of cutaneous lupus has not been studied.

**Objective:**

It was to assess the levels of a selected panel of circulating miRNAs that could be involved in the regulation of the immune response, inflammation, and fibrosis in cutaneous lupus.

**Methods:**

It was a cross-sectional study. We included 22 patients with subacute (SCLE) and 20 with discoid (DLE) lesions, and 19 healthy donors (HD). qRT-PCR for miRNA analysis, flow cytometry in peripheral blood, and skin immunohistochemistry were performed to determine the distribution of CD4 T cells and regulatory cells and their correlation with circulating miRNAs.

**Results:**

miR-150, miR-1246, miR-21, miR-23b, and miR-146 levels were downregulated in SCLE *vs.* HD. miR-150, miR-1246, and miR-21 levels were downregulated in DLE *vs.* HD. miR-150, miR-1246, and miR-21 levels were downregulated in DLE *γ*^+^ with miR-1246 in SCLE, whereas CD123^+^/CD196^+^/IDO^+^ cells were positively associated with miR-150 in DLE. In the tissue, CD4^+^/IL-4^+^ and CD20^+^/IL-10^+^ cells were positively associated with miR-21 and CD4^+^/IFN-*γ*^+^ with miR-1246 in SCLE, whereas CD123^+^/CD196^+^/IDO^+^ cells were positively associated with miR-150 in DLE. In the tissue, CD4^+^/IL-4^+^ and CD20^+^/IL-10^+^ cells were positively associated with miR-21 and CD4^+^/IFN-*β*, thyroid hormone, and cancer signaling pathways were shared between miR-21, miR-31, miR-23b, miR-146a, miR-1246, and miR-150.

**Conclusions:**

A downregulation of miR-150, miR-1246, and miR-21 in both CLE varieties *vs.* HD. miR-150, miR-1246, and miR-21 levels were downregulated in DLE

## 1. Introduction

Cutaneous lupus erythematosus (CLE) is an autoimmune condition that comprehends a wide range of dermatological manifestations and clinical phenotypes. Its pathogenesis is multifactorial and involves genetic predisposition, environmental factors (ultraviolet light B), and abnormalities in the innate and adaptive immune response. In this context, the participation of some proinflammatory cytokines such as IFN-*α*, IL-1, IL-6, and TNF-*α* has been also recognized [1, 2]. Histological analysis of CLE skin is characterized by a dense periadnexal and perivascular lymphocytic infiltrate, mainly composed by CD4^+^ helper T cells, CD8^+^ cytotoxic T cells, B cells, and macrophages [1]. Moreover, the participation of Th17 and Th22 CD4^+^ T cells as well as regulatory T cells has been recently described [3, 4]. In addition, the activation of pathways via pattern recognition receptor (PRR) signaling, Janus kinase- (JAK-) signal transducer and activator of transcription (STAT) signaling, and nuclear factor-*κ*B (NF-*κ*B) signaling also have a role in CLE [5].

Recent studies have revealed the potential contribution of microRNAs (miRNAs) in diverse autoimmune diseases [6]. miRNAs are a class of small noncoding RNAs that modulate gene expression at the posttranscriptional level. They bind to the target messenger RNA, leading either to translational repression or to degradation. miRNAs regulate diverse physiologic processes, and their dysregulation can result in aberrant responses including impaired immune function [6–8].

For instance, in systemic lupus erythematosus (SLE), miRNA expression has been detected in the plasma [9], serum [10], urine, and peripheral blood mononuclear cells (PBMCs) [11]. Diverse miRNA patterns have been associated with a certain feature such as renal involvement (miR-146a), childhood onset (miR-516a-3p, miR-629, and miR-525-5p), and overall disease activity (miR-21 and miR-146a) [9–11] and recently, in discoid lupus (DLE) (miR-31 and miR-485-3p) [12]. Indeed, a study suggested miR-29b as a potential SLE diagnostic biomarker. The authors in that study, using a ROC curve analysis, showed an AUC of 0.75 (95% CI 0.64–0.86) for diagnosing SLE [13].

On the other hand, research focused on understanding the role of miRNAs in the regulation of signaling pathways may lead to develop new biomarkers and new therapeutic approach. In this sense, circulating miR-29b was also proposed as a biomarker to estimate lupus activity as it positively correlated with the SLEDAI score and anti-dsDNA titer and inversely correlated with complement C3 level and clinical response after treatment [13].

Herein, we hypothesized that the expression of a circulating miRNA signature might distinguish CLE patients and its subtypes. Therefore, our main objective was to evaluate the circulating levels of a selected panel of miRNAs based on their possible participation in the regulation of the immune response, inflammation, and fibrosis among patients with CLE. Specifically, we evaluated the following miRNAs: miR-29 family that regulates T cell polarization [14]; miR-150 involved in B and Th17 cell differentiation and TGF-*β* signaling [15]; miR-23b related to IL-17, TNF-*α*, and IL-1*β* expression [16]; miR-1246 which is associated to B cell activation [17]; miR-21 inked with Th2 and Th17 differentiation and Foxp3^+^ expression [18]; miR-31 that regulates Treg cells, NF-*κ*B activation, and fibrosis [19]; miR-146 that appears to downregulate NF-*κ*B and TLR/MyD88 proinflammatory signal pathways [20, 21]; miR-155 that contributes to Th1 and Th17 differentiation [22]; miR-485 that participates in Th2 differentiation [12]; and miR-197 that allows the IL-22 responses [23]. Moreover, we also correlated these serum miRNAs with T, B, and regulatory cell subpopulations in the skin tissue and peripheral blood as well as with the CLASI activity score. Finally, we elucidated their possible participation in CLE pathogenesis applying bioinformatics.

## 2. Materials and Methods

### 2.1. Patients

This was a cross-sectional study conducted in a tertiary care center. We included 42 consecutive patients with CLE: 22 with subacute cutaneous lupus (SCLE) and 20 with DLE. To be eligible, patients also had to meet the classification criteria for SLE according to the ACR criteria [24] and to have an active lupus-specific lesion compatible with SCLE or DLE. The diagnosis of CLE was established in consensus by a rheumatologist and a dermatologist, as well as by biopsy. In addition, patients should not be under topical treatment including steroids within the last 6 weeks. However, patients could maintain their basal oral steroids and immunosuppressants. Patients were excluded if they had any concomitant cutaneous lesion not attributed to lupus or an overlap autoimmune condition.

We included 19 healthy donors (HD) as controls. The control group did not have any autoimmune disease and/or concurrent infection and did not receive prednisone or immunosuppressants.

We measured the CLASI, a validated index to quantify disease severity [25]. As this instrument measures both activity and damage, for the present study, we only used the activity domain that ranges from 0 to 70 (higher scores are indicative of more severity).

In addition, patients' clinical records were carefully reviewed according to a preestablished protocol to collect demographics as well as other clinical and serologic features.

### 2.2. Skin Samples

Skin punch biopsies (4 mm diameter) were performed, fixed in formalin, and evaluated with hematoxylin-eosin staining for the assessment of classic histologic cutaneous lupus features. Then, the rest of the specimen was stored for immunohistochemistry. Microscopic review was performed in a blinded manner to the diagnosis by one blinded observer.

Overall, most of the cutaneous lupus biopsies corresponded to photoexposed areas localized at the arms, thorax, or scalp. Control tissue biopsies were also taken from photoexposed areas and, if possible, from the same anatomical zone.

### 2.3. Immunohistochemistry

We followed the methods of Méndez-Flores et al. [26]. Briefly, IL-22-expressing cells were determined in 4 *μ*m thick sections of tissue. After deparaffinization and demasking of antigens, tissues were blocked with 3% H_2_O_2_. Then, nonspecific background staining was avoided with the IHC background blocker (Enzo Life Sciences). Tissues were incubated with goat polyclonal anti-human IL-22 antibody (Santa Cruz Biotechnology, Santa Cruz, CA, USA) at 10 *μ*g/ml. Binding was identified with biotinylated donkey anti-goat IgG antibody (ABC Staining System; Santa Cruz Biotechnology). Slides were incubated with horseradish peroxidase- (HRP-) streptavidin, followed by incubation with the peroxidase substrate 3,3-diaminobenzidine (DAB) (Sigma-Aldrich) for 10 min. The sections were counterstained with hematoxylin. Negative control staining was performed with normal human serum diluted 1 : 100, instead of primary antibody, and the IHC universal negative control reagent (IHC universal negative control reagent, Enzo Life Sciences). The reactive blank was incubated with phosphate buffer saline-egg albumin (Sigma-Aldrich) instead of the primary antibody. Both controls excluded nonspecific staining or endogenous enzymatic activities [26]. Spleen and ganglion samples were used as a positive control ([Supplementary-material supplementary-material-1]).

### 2.4. Double-Staining Procedure

We followed the methods of Méndez-Flores et al. [26]. To determine the subpopulation of CD4^+^/IL-17A^+^–, CD4^+^/IL-4^+^–, CD4^+^/IFN-*γ*^+^-expressing T cells, CD25^+^/Foxp3^+^ regulatory T cells, CD20^+^/IL-10^+^-producing B cells, and CD123^+^/IDO^+^ pDC cell subpopulations, a simultaneous detection was performed (MultiView (mouse-HRP/rabbit-AP) Enzo Life Sciences). After deparaffinization and demasking of antigens with the antigen retrieval reagent (Enzo Life Sciences), tissues were blocked with 3% H_2_O_2_. The procedure is a sequential double staining where the first antigen (normal serum as negative control, rabbit polyclonal anti-IL-17A, anti-IL-4, anti-IFN-*γ*, anti-IDO IgG antibody, or mouse monoclonal anti-IL-10 or anti-Foxp3 IgG_1_ antibody (Santa Cruz Biotechnology) at 10 *μ*g/ml) was visualized using horseradish peroxidase (HRP)/3′3′-diaminobenzidine (DAB) and the second antigen (normal serum as negative control or second primary rabbit polyclonal anti-CD20, anti-CD25 IgG antibody or mouse monoclonal anti-CD4, anti-IgG_1_ antibody (Santa Cruz Biotechnology), or anti-CD123 IgG antibody (Abcam pcl, CA, UK) at 10 *μ*g/ml) was visualized using alkaline phosphatase (AP)/Permanent Red. Tissues were counterstained with Mayer's hematoxylin and mounted in aqueous mounting medium. Cytokine-expressing cells as well as double positive cells were assessed by estimating the number of positively staining cells in two fields (X320) and were reported as the percentage of immunoreactive cells of the inflammatory infiltrates located at the epidermis and dermis. Results are expressed as the mean ± standard error of the mean (SEM) of cells quantified by the program Image Pro Plus version 5·1·1 [26].

### 2.5. Peripheral Blood Samples

A venous blood sample was drawn from each subject to perform flow cytometry analysis and RNA isolation.

### 2.6. Flow Cytometry

Peripheral blood mononuclear cells (PBMCs) were obtained by gradient centrifugation on Lymphoprep (Axis-Shield PoC AS, Oslo, Norway). Cell pellet was resuspended in 1 ml RPMI at 1‐2 × 10^6^ cell/ml, and cell suspension was treated with 2 *μ*l of a cell activation cocktail of phorbol-12myristate 13-acetate (40.5 *μ*M) and ionomycin (669.3 *μ*M) in DMSO (500x) and brefeldin A (BioLegend Inc., San Diego, CA, USA) for 6 hours at 37°C in a CO_2_ incubator.

PBMCs were incubated with 5 *μ*l of Human TruStain FcX™ (BioLegend Inc.) per million cells in 100 ml PBS for 10 minutes, and then, they were labeled with 5 *μ*l of antihuman CD3-FITC-labeled, antihuman CD4-PeCy5-labeled, and antihuman CD161-APC-conjugated monoclonal antibodies (BD Biosciences, San Jose, CA); antihuman CD3-FITC-labeled, antihuman CD4-PeCy5-labeled, and antihuman CD25-APC-conjugated monoclonal antibodies (BD Biosciences); antihuman CD19-APC-labeled, antihuman CD24-FITC-conjugated, and antihuman CD38-PeCy5-labeled monoclonal antibodies (BD Biosciences); or antihuman CCR6-PerCP/Cy5.5-conjugated and antihuman CD123-FITC-labeled monoclonal antibodies (BD Biosciences) in separated tubes during 20 min at 37°C in the dark. Cells were permeabilized with 200 *μ*l of cytofix/cytoperm solution (BD Biosciences) at 4°C for 30 min. Intracellular staining was performed with an anti-human IL-22-PE-, IL-17A-PE-, IL-4-PE-, IFN-*γ*-PE-, Foxp3-PE-, IL-10-PE-, and IDO-PE-labeled mouse monoclonal antibodies (BD Biosciences) for 30 min at 4°C in the dark. An electronic gate was made for CD3^+^/CD4^+^/CD161^−^ cells, CD3^+^/CD4^+^/CD161^+^ cells, CD3^+^/CD4^+^/CD25^−^ cells, CD3^+^/CD4^+^/CD25^hi^ cells, CD19^+^/CD38^hi^/CD24^hi^ cells, and CD123^hi^/CD196^+^ cells. Results are expressed as the relative percentage of IL-22^+^, IL-17A^+^, IL-4^+^, IFN-*γ*^+^, Foxp3^+^, IL-10^+^, and IDO^+^ expressing cells in each gate. As isotype control, IgG1-FITC/IgG1-PE/CD45-PeCy5 mouse IgG1 *kappa* (BD Tritest, BD Biosciences) was used to set the threshold and gates in the cytometer. We ran an unstained (autofluorescence control) and permeabilized PBMC sample. Autofluorescence control was compared to single-stained cell-positive controls to confirm that the stained cells were on scale for each parameter. Besides, BD Calibrate 3 beads were used to adjust instrument settings, set fluorescence compensation, and check instrument sensitivity (BD calibrates, BD Biosciences). Fluorescence minus one (FMO) controls were stained in parallel using the panel of antibodies with sequential omission of one antibody, except for the anti-IL-22, anti-IL-17A, anti-IL-4, anti-IFN-*γ*, anti-Foxp3, anti-IL-10, and anti-IDO antibody, which was replaced by an isotype control rather than simply omitted. Finally, T subsets were analyzed by flow cytometry with an Accuri C6 (BD Biosciences). A total of 500,000–1,000,000 events were recorded for each sample and analyzed with the FlowJo X software (Tree Star, Inc.) [26].

### 2.7. RNA Isolation and Quantitative Real-Time PCR Analyses of MicroRNAs

Total serum RNA was isolated with TRIzol reagent (Invitrogen) according to the manufacturer's instructions. The concentration and quality of total RNA were measured by a NanoDrop 1000 Spectrophotometer (NanoDrop Technologies, Waltham, Mass). To quantify miRNAs, cDNA was synthesized using a Mir-X miRNA first-strand synthesis kit (Clontech) according to the manufacturer's instructions. Complementary DNA (cDNA) was amplified by real-time PCR with a SYBR green-based fluorescent method using the Maxima SYBR Green/ROX qPCR Master Mix (2X) (Thermo Scientific). *U6* was used as an endogenous control to normalize the expression values. We chose the following panel of miRNAs, for their effect reported in the literature in the different regulatory cells studied here. Primer sequences used for real-time PCR were as follows: hsa-miR-21-5p, 5′-TAGCTTATCAGACTGATGTTGA-3′; hsa-miR-29a, 5′-TAGCACCATCTGAAATCGGTTA-3′; hsa-miR-29b, 5′-TAGCACCATTTGAAATCAGTGTT-3′; hsa-miR23b, 5′-TGGGTTCCTGGCATGCTGATTT-3′; hsa-miR-31, 5′-AGGCAAGATGCTGGCATAGCT-3′; hsa-miR-146a, 5′-TGAGAACTGAATTCCATGGGTT-3′; hsa-miR-155, 5′-TTAATGCTAATCGTGATAGGGGT-3′; hsa-miR-150, 5′-TCTCCCAACCCTTGTACCAGTG-3′; hsa-miR-1246, 5′-AATGGATTTTTGGAGCAGG-3′; hsa-miR-197-3p, 5′TTCACCACCTTCTCCACCCAGC-3′; hsa-miR-485-p, 5′AGAGGCTGGCCGTGATGAATTC-3′; and U6 forward, 5′-GCTTCGGCAGCACATATACTAAAAT-3′ and U6 reverse, 5′-CGCTTCACGAATTTGCGTGTCAT-3′.

qRT-PCR assays were performed in a StepOne real-time PCR instrument (Life Technologies, Foster City, CA). The reactions were carried out with a 10 min incubation at 95°C followed by 40 cycles of 95°C for 15 s and 60°C for 1 min. All reactions were run in triplicate, and the average threshold cycle and SD values were calculated. The transcript levels were calculated based on the threshold cycle (C_t_) using the delta-delta C_t_ method that measures the relative of a target RNA between two samples by comparing them to a normalization control RNA (*U6*).

### 2.8. Target Gene and Pathway Enrichment Analysis

We determined the gene targets of identified miRNAs using DIANA-TarBase v8 (http://www.microrna.gr/tarbase), which is a database containing experimentally validated miRNA-target interactions. We used DIANA-miRPath v3.0 (http://www.microrna.gr/miRPathv3) and the Kyoto Encyclopedia of Genes and Genomes (KEGG) database for the identification of the networks and pathway enrichment of the selected miRNA target genes. Enriched pathways showing statistical significance (*P* ≤ 0.05) were subjected to further molecular analysis and interactome network construction. Interactome networks were constructed to connect miRNAs to their putative target genes (or to enrichment pathways) within the selected enriched pathways; the resulting networks were exported to Cytoscape v3.1.0 (http://cytoscape.org/index.php) for visualization.

### 2.9. Ethical Considerations

This work was performed according to the principles expressed in the Declaration of Helsinki. The study was approved (Reference 822) by the Ethical Committee from the Instituto Nacional de Ciencias Médicas y Nutrición Salvador Zubirán, and a written informed consent was obtained from all subjects.

### 2.10. Statistical Analysis

Descriptive statistic was performed, and categorical variables were compared using the Chi-2 test or Fisher's exact test. We used Mann-Whitney *U*-test for comparison of two medians. One-way analysis of variance on ranks Kruskal-Wallis, if the Kruskal-Wallis test was significant, a post hoc analysis (Dunn's test) was performed for all pairwise multiple comparison procedures. The relative expression of miRNAs was reported as medians and ranges (5th/95th percentiles). We reported nonparametric correlations using Spearman coefficients among serological relative expression of miRNAs and CLASI score. A strong correlation was defined as a Spearman coefficient between ±0.50 and ±1, a medium correlation between ±0.30 and ±0.49, and a weak correlation below 0.29. We also performed a multiple linear regression analysis to evaluate the possible association of the meaningful miRNAs and the peripheral and skin cell subpopulations among CLE varieties.

All the statistical tests were performed 2-sided; *P* values less than 0.05 were considered statistically significant. SPSS (v. 21.0) and GraphPad Prism (v. 5) software was used for statistical analysis.

## 3. Results

### 3.1. Clinical and Demographic Characteristics

Clinical and demographic characteristics of the groups are summarized in Table 1. Most of the participants were females and had a similar age. We did not find significant differences regarding SLE duration, prednisone dose, and use of antimalarials and immunosuppressants among the groups with CLE. However, as expected, the median of the CLASI activity score in SCLE patients was higher than the score of DLE patients.

### 3.2. miRNA Profiling in SCLE and DLE Patients

**Figure 1:**
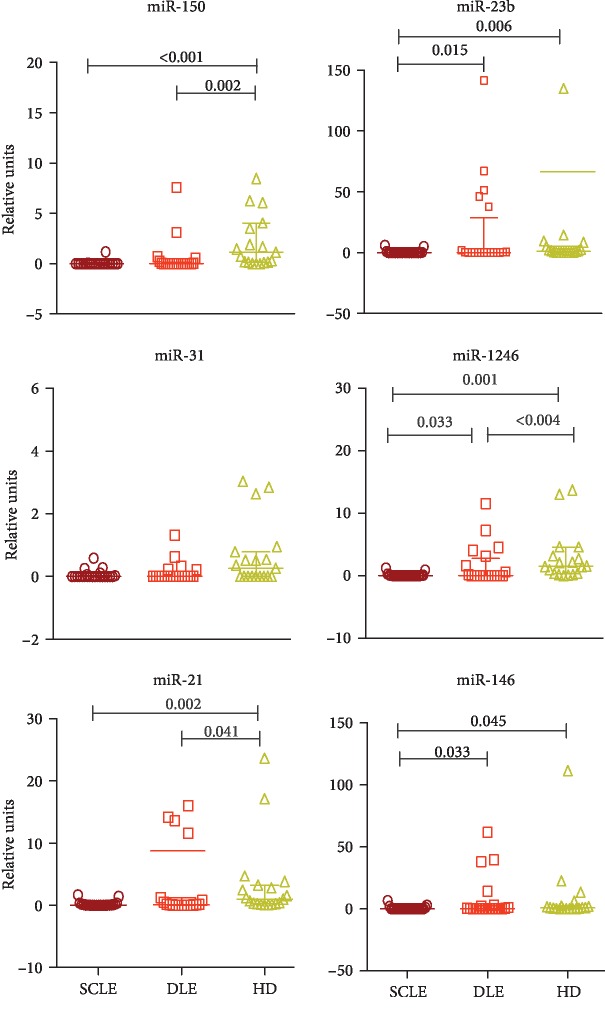
Expression of differentially expressed miRNAs in cutaneous lupus. ^∗^The results are expressed as the median and range (5th/95th percentiles). Kruskal-Wallis test and post hoc analysis (Dunn's test). SCLE: subacute cutaneous lupus erythematosus; DLE: discoid lupus erythematosus; HD: healthy donors.

The analysis revealed differential circulating levels of six miRNAs (miR-150, miR-23b, miR-1246, miR-21, miR-31, and miR-146) among the patients' groups and controls (Figure 1). In this regard, circulating levels of miR-1246 (372.7-fold decrease), miR-150 (183.1-fold decrease), miR-21 (14.7-fold decrease), miR-23b (14-fold decrease), and miR-146 (13-fold decrease) were considerably lower in patients with SCLE than in healthy controls. Patients with DLE also had lower levels of miR-21 (4.6-fold decrease), miR-1246 (3.8-fold decrease), and miR-150 (1.7-fold decrease) than healthy controls. When we compared the group with SCLE versus DLE, the SCLE group had lower levels of miR-1246 (75.5-fold decrease), miR-146 (40.6-fold decrease), and miR23b (23.2-fold decrease).

### 3.3. Correlation between miRNAs and CLASI Activity Score

Except for miR-150 in the subgroup of patients with SCLE (*ρ* = −0.64 CI 95% -0.78 to -0.11, *P* = 0.01), we did not find any correlation among the CLASI activity score and other circulating miRNA levels.

### 3.4. Associations of miRNAs and Circulating and Skin Cell Subpopulations

We performed a multiple regression analysis to evaluate the association of the different miRNAs and the circulating and skin cell subsets.

**Figure 2:**
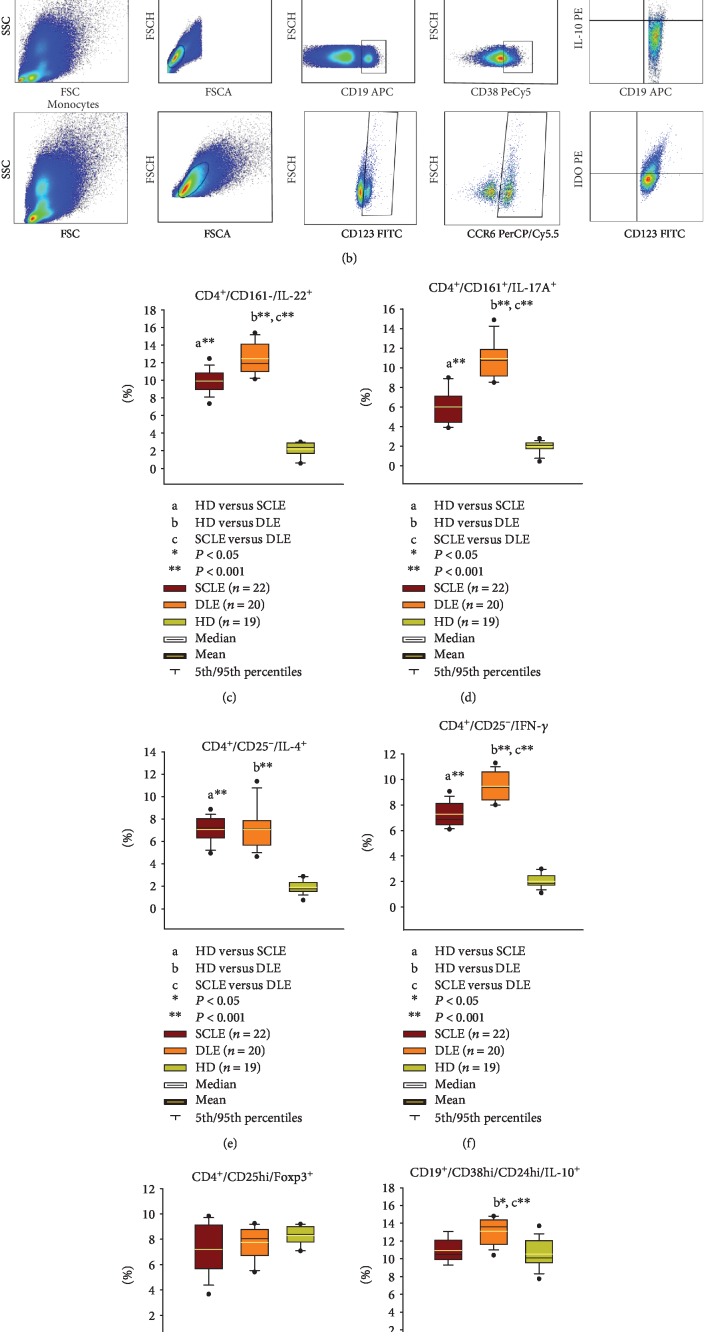
(a) Representative gating strategy of each cell population of CD4 effector T cells. (b) Representative gating strategy of each cell population of regulatory cells of a DLE patient. Percentages of circulating (c) CD4^+^/CD161^−^/IL-22^+^ cells, (d) CD4^+^/CD161^+^/IL-17A^+^ cells, (e) CD4^+^/CD25^−^/IL-4^+^ cells, (f) CD4^+^/CD25^−^/IFN-*γ*^+^ cells, (g) CD4^+^/CD25hi/Foxp3^+^ cells, (h) CD19^+^/CD38hi/IL-10^+^ cells, and (i) CD123^+^/CD196^+^/IDO^+^ cells. The results are expressed as the mean (horizontal yellow line), median (horizontal black line), and 5th/95th percentiles ^∗^*P* < 0.05 and ^∗∗^*P* < 0.001. SCLE: subacute cutaneous lupus erythematosus; DLE: discoid lupus erythematosus; HD: healthy donors.

At peripheral blood, in the group of SCLE, we observed a negative association with CD4^+^/CD25^−^/IL-4^+^ cells and CD4^+^/CD25^hi^/Foxp3^+^ and miR-23b (Figures 2(a), 2(e), and 2(g), Table 2) and CD4^+^/CD25^−^/IFN-*γ*^+^ with miR-1246 (Figures 2(a) and 2(f), Table 2). In the group of DLE patients, there was a positive association with CD123^+^/CD196^+^/IDO^+^ plasmacytoid dendritic cells with miR-150 (Figures 2(b) and 2(i), Table 3).

**Figure 3:**
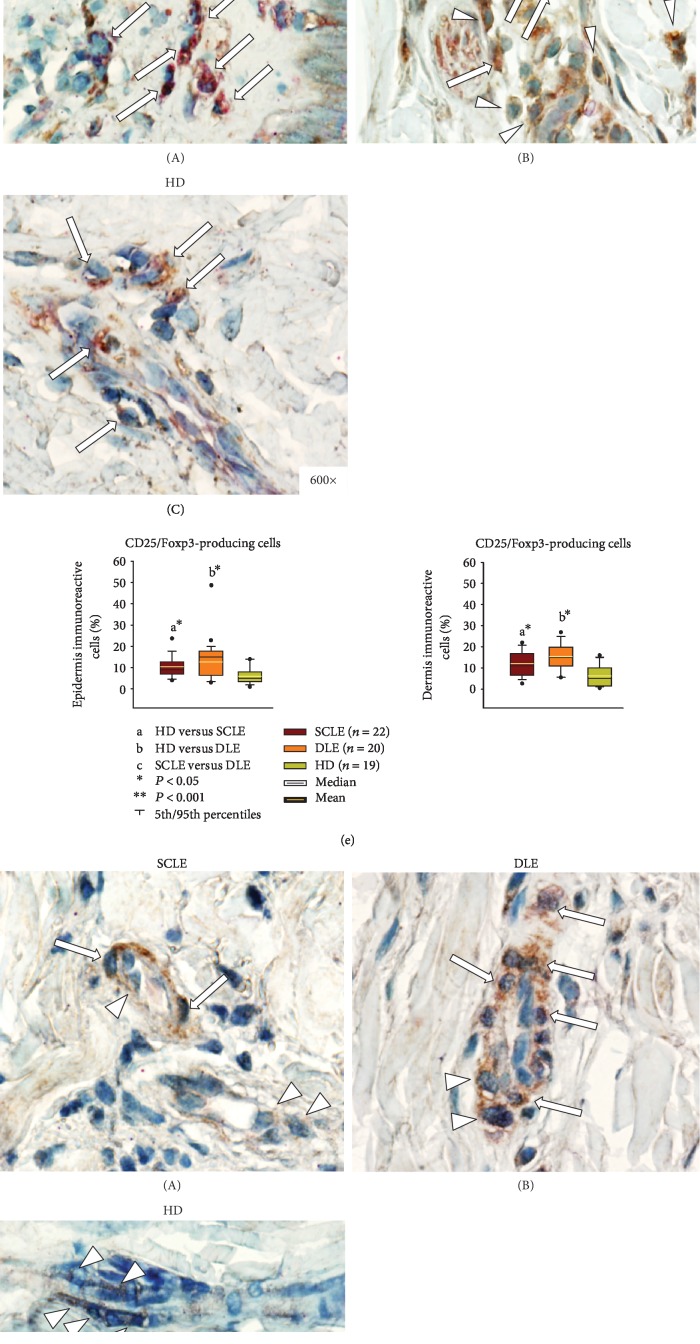
Representative immunostaining of (a) IL-22-expressing cells, (b) IL-17A-expressing CD4 T cells, (c) IL-4-expressing CD4 T cells, (d) IFN-*γ*-expressing CD4 T cells, (e) Foxp3-expressing CD25 T cells, (f) IL-10-expressing CD20 B cells, and (g) IDO-expressing CD123 plasmacytoid dendritic cells in tissue biopsies from subacute cutaneous lupus erythematosus ((A) SCLE), discoid lupus erythematosus ((B) DLE), healthy donors ((C) HD). Arrowheads show single staining (in brown or red), and arrows depict double staining (in burgundy). Original magnification was ×600. (h–n) Percentage of immunoreactive cells per microscopic field. The results are expressed as the mean (horizontal yellow line), median (horizontal black line), and 5th/95th percentiles. ^∗^*P* < 0.05 and ^∗∗^*P* < 0.001. SCLE: subacute cutaneous lupus erythematosus; DLE: discoid lupus erythematosus; HD: healthy donors.

At the skin, in the group of SCLE, a positive association was determined between miR-21 and CD4^+^/IL-4^+^ (Figure 3(c), Table 2); and CD20^+^/IL-10^+^ (Figure 3(f), Table 2) and CD4^+^/IFN-*γ*^+^ with miR-31 (Figure 3(d), Table 2). In the DLE patients' group, CD4^+^/IL-4^+^ cells were associated positively with miR-150 (Figure 3(c), Table 3), and CD20^+^/IL-10^+^ cells with miR-1246 and miR-146a (Figure 3(f), Table 3).

### 3.5. Kyoto Encyclopedia of Genes and Genomes (KEGG) Pathway Enrichment Analysis of miRNAs Differentially Expressed in Patients with SCLE and DLE

The predicted targets and pathways of the differentially expressed miRNAs and their correlation with specific circulating and skin T cell subpopulations in both clinical conditions (SCLE: miR-21, miR-31, and miR-23b and DLE: miR-150, miR-1246, and miR-146a) were analyzed by using the KEGG pathway enrichment strategy. The analysis of the predicted targets and pathways revealed that in SCLE, miR-21 was linked with Hippo signaling, bacterial invasion of epithelial cells, transcriptional regulation of cancer, prolactin signaling, FoxO signaling, and biosynthesis and degradation of fatty acids. Interestingly, miR-31 also was connected with cancer pathways associated with miR-21 and miR-23b. In addition, we found that miR-31 was significantly associated with TNF signaling.

Regarding DLE, miR-146a expression was significantly linked to immune relevant pathways including NF-kappa B signaling, Toll-like receptor signaling. miR-1246 was associated with apoptosis and viral carcinogenesis. Finally, miR-150 in CLE was closely related to HIF-1 signaling and cancer pathways.

**Figure 4:**
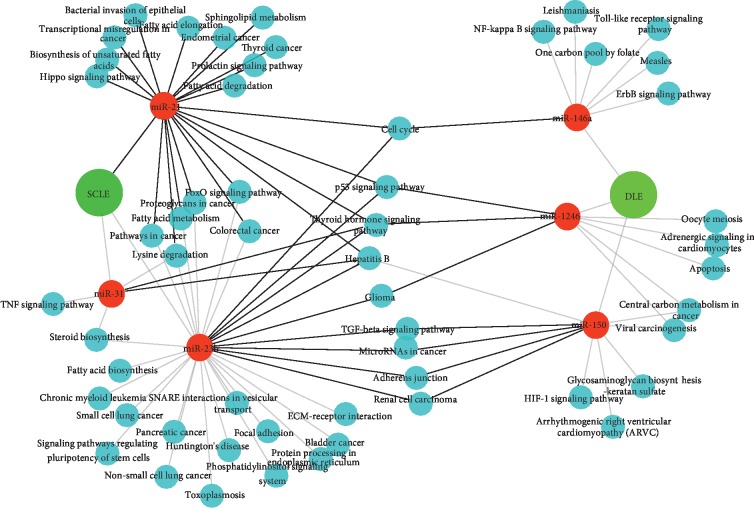
Interactome analysis of the most significantly enriched networks of differentially expressed miRNAs in SCLE and DLE patients. miR-21, miR-31, and miR-23b (red circles) are strongly associated with SCLE whereas miR-146a, miR-1246, and miR-150 (red circles) are linked with DLE. Significantly associated pathways enriched in KEGG analysis of these miRNA signatures are also shown in dark green circles.

Interestingly, the interactome analysis showed a group of pathways that were linked with specific miRNAs expressed in SCLE and DLE patients. In this context, cell cycle regulation, p53 signaling, TGF-*β* signaling, thyroid hormone signaling, and cancer pathways were shared between miR-21, miR-31, and miR-23b (expressed in SCLE) and miR-146a, miR-1246, and miR-150 expressed in DLE patients (Figure 4).

**Figure 5:**
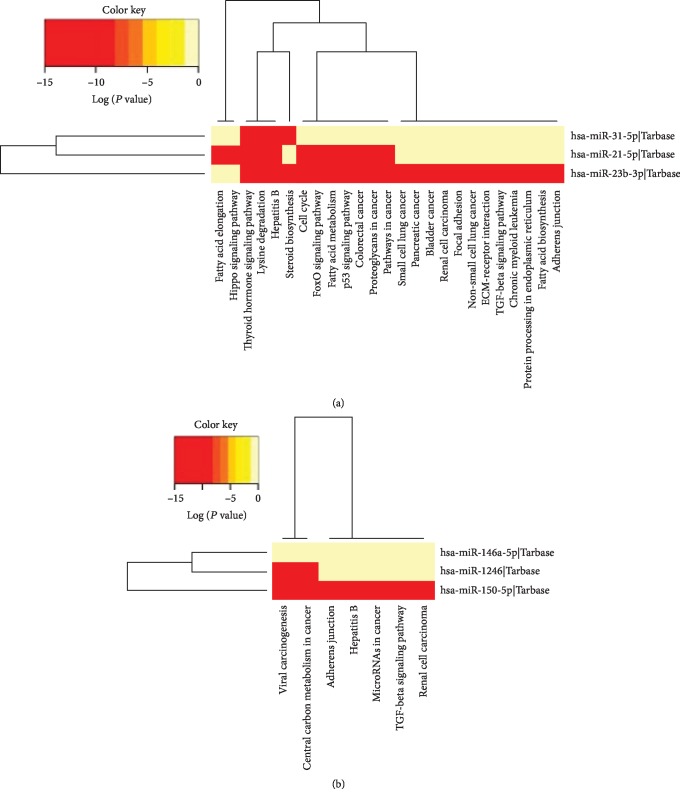
(a) Heat map of KEGG pathways enriched with gene regulated by a combination of hsa-miR-23b-3p, hsa-miR-21-5p, and hsa-miR-31-5p (SCLE); (b) heat map of KEGG pathways enriched with gene regulated by a combination of hsa-miR-150-5p, hsa-miR-1246, and hsa-miR-146a-5p (DLE).

Importantly, the degree of association between the differentially expressed miRNA signatures and specific T cell subpopulations observed in SCLE and DLE patients and their enrichment with the aforementioned pathways are shown in Figure 5. Detailed *P* values and the degree of association of specific miRNAs and functional pathways are enlisted in Figures [Supplementary-material supplementary-material-1] and [Supplementary-material supplementary-material-1].

## 4. Discussion

miRNAs are key determinants in the posttranscriptional regulation of genome [27, 28] and are involved in the control of many biological processes including inflammation [29]. It is well known that an uncontrolled immune inflammatory response might contribute to the pathogenesis of autoimmunity.

In the present work, we explored if the expression of circulating miRNA signature might identify patients with CLE and their varieties, as was described by Solé et al. for miR-31 and miR-485-3p overexpression in DLE compared to SCLE [12]. Our most relevant findings were that miR-150, miR-1246, miR-21, miR-23b, and miR-146 were downregulated in SCLE than in healthy controls. Furthermore, miR-1246, miR-23b, and miR-146 expression was lower in SCLE than in DLE. We observed some differences regarding the association of peripheral and tissue subpopulation expression and the miRNAs according to CLE varieties. Notwithstanding in the SCLE group, miR-23b and miR1246 drove a Th2 and Th1 peripheral response; whereas in the DLE group, miR-1246 was associated with infiltrates of IL-10-producing B cells.

miR-150 is expressed in different lineages of immune cells and is involved in cellular maturation from pro-B to pre-B lymphocytes, as well as in the development and functional activity of NK and iNKT cell lineages [30, 31]. Regarding the participation of miR-150 at the skin biology, it has been demonstrated that its downregulation promotes keratinocyte proliferation in hypoxic conditions through targeting HIF-1*α* and VEGF-A [32]. Besides, miR-150 downregulation is associated with the constitutive type I collagen overexpression in scleroderma dermal fibroblasts via the induction of integrin *β*_3_ [33]. miR-150 seems to play an important role in the induction of myofibroblast proliferation and its resistance to apoptosis [34]. Moreover, miR-150 is also downregulated in other autoimmune diseases with skin involvement, including psoriasis and diffuse cutaneous systemic sclerosis (SSc) skin lesions [32]. Specifically, in SSc, downregulation promotes TGF-*β* signaling and Smad3 phosphorylation, resulting in the transcriptional activation of type I collagen gene and tissue fibrosis [32]. In this vein, a study described that patients with DLE exhibited a distinctive overexpression signature of profibrotic markers including TGF-*β* and SMAD3 [12]. Also, in line with these studies, our findings suggest that the downregulation of miR-150 in CLE might influence the activation of chronic inflammatory and profibrotic pathways.

On the other hand, the role of miR-1246 in normal skin and autoimmune disorders is still poorly characterized. In patients with SLE, it was found downregulated. This low expression might be related to commitment hyperactivation of B cells. Interestingly, B cells from nonactive SLE patients have similar expression levels of miR-1246 when compared with healthy volunteers [17]. Moreover, it has been demonstrated that miR-1246 promotes UVB-induced apoptosis by downregulating RTKN2 expression in keratinocytes [35]. Herein, we observed that this miRNA was also downregulated in patients with SCLE and DLE.

Concerning miR-146, it has been observed that melanoma lesions overexpress it [36]. In patients with psoriasis, miR-146a is positively correlated with IL-17 expression in skin lesions and PBMCs [37]. Furthermore, miR-146a regulates the proinflammatory TLR/MyD88 pathway by targeting IRAK1 and TRAF6 [38]. miR-146a also contributes to the development of SLE, due to the reason that it is a negative regulator of type I IFN pathway by targeting IRF5, STAT1, IRAK1, and TRAF6 [34]. As in patients with SLE, herein, we observed a downexpression in CLE. Thus, the decreased expression of miR-146a in PBMCs might contribute to the enhanced production of type I IFN (IFN-*α*/*β*) in human lupus.

miR-23b, which is a differentiation marker of human keratinocytes (through repression of TGIF1 and activation of the TGF-*β*-SMAD2 signaling pathway), is remarkably upregulated after UVA irradiation of human primary keratinocytes and acts through targeting-related RAS viral oncogene homolog 2 (RRAS2), which is strongly expressed in highly aggressive malignant skin cancer [39, 40]. Moreover, miR-23b promotes cutaneous wound healing through inhibition of the inflammatory responses by targeting ASK1 [41].

Furthermore, we reported a downexpression of miR-21 among CLE patients but not of miR-31. miR-21, as well as miR-31, are master regulators of T cell activation in SLE [42]. Several studies have demonstrated that aberrant expression of miR-21 is involved in the pathogenesis of SSc and psoriasis. miR-21 regulates genes such as SMAD3, SMAD7, and type I collagen, all involved in fibrosis [34]. Moreover, it has been demonstrated a reciprocal regulation between thyroid hormone and miR-21 where miR21 downregulates hedgehog pathway-driven skin tumorigenesis (basal cell carcinoma) [43]. Overexpression of miR-21 also inhibits the growth and metastasis of melanoma cells by targeting MKK3 [44].

In SLE patients, miR-31 was downregulated and negatively associated with disease activity and proteinuria, whereas miR-21 high expression correlated with the SLEDAI score and proteinuria. In addition, a reduced expression of miR-31 appears to alter the production of IL-2 by T cells in SLE patients, possibly influencing the expression of nuclear factor of activated T cells (NFAT) [45].

On the other hand, a recent study described a specific miRNA skin signature for DLE that included miR-31 and miR-485-3p. It demonstrated that the overexpression of miR-31 was stimulated by UV and TGF-*β* and this miRNA activated NF-*κ*B signaling stimulating proinflammatory responses resulting in neutrophil and macrophage infiltrates in skin [2]. miR-31 is a key regulator for promoting keratinocyte proliferation and migration during wound healing [46], and it is overexpressed in SSc, a systemic disease characterized by extensive fibrosis [47, 48].

Finally, the participation of some immune response pathways including PRR signaling, JAK-signal transducer, STAT signaling, NF-*κ*B signaling, and mitogen-activated protein kinase (MAPK) signaling cascade has been described in CLE [5]. Herein, several pathways were linked with the circulating miRNAs differentially expressed in CLE. Interactome network analysis revealed a strong connection between circulating miRNAs in SCLE and DLE groups with a particularly solid network connection such as cell cycle regulation pathways, p53 signaling, TGF-*β* signaling, NF-*κ*B signaling, HIF-1 pathway, thyroid hormone signaling, and cancer pathways, among others. Some of them, as previously mentioned, had been described in CLE, but others did not, opening new areas of research. Overall, new insights into the pathogenesis of CLE might allow the development of new target treatments such as the inhibition of these immune pathways.

Certainly, we acknowledge that our study has the following limitations: first, a limited sample size and the lack of a replication cohort. Notwithstanding, our sample was carefully clinically selected and was able to detect differences in the expression of miRNAs among the different groups of CLE patients. Second, we did not evaluate the presence of miRNAs in skin biopsies. However, we were interested in studying circulating miRNAs with the purpose of further using them as future potential blood-based biomarkers to assess relapses and response to treatment, without the need of a skin biopsy. Finally, at the analysis of peripheral and skin subpopulations, none of the downregulated circulating miRNAs were associated with Th17 and Th22 cells previously recognized as participants in CLE pathogenesis. Thus, it is highly probable that other miRNAs might be also implicated in the regulation of these cell subpopulations. Besides these limitations, we consider that our current manuscript is of relevance particularly for the scant information regarding the pathogenesis of CLE.

Summing up, we determined downregulation of miR-150, miR-1246, and miR-21 in CLE patients. SCLE variety had the lowest levels of miR-1246, miR-146, and miR-23b. Overall, these miRNAs drove the presence of different peripheral and skin subpopulations and participated in diverse pathways. Further research is needed to validate our results in other populations and to evaluate the role of these miRNAs as clinical biomarkers.

## Figures and Tables

**Table 1:** Clinical characteristics from patients with cutaneous lupus erythematosus.

	Cutaneous lupus erythematosus		Healthy donor	*P* value
	SCLE *n* = 22	DLE *n* = 20	*n* = 19	
*Demographics*				
Age (years)				
Mean ± SD	38.2 ± 13.6	37.4 ± 15.3	43.6 ± 16.5	
Median	34.5	32.5	45.0	
Range	(20-62)	(18-74)	(21-76)	
Sex, females, (%)	95.5	95	95	
Disease duration (years)				
Mean ± SD	10.2 ± 10.3	7.5 ± 6.4	–	
Median	6.0	7.0		
Range	(1-41)	(1-26)		
*Laboratory variables*				
Hemoglobin (g/dl)				
Mean ± SD	12.6 ± 2.0	13.4 ± 1.5	14.4 ± 1.0	
Median	13.0	13.2	14.5	
Range	(9.3–15.8)	(11.2–16.5)	(12.5–15.8)	
Leucocytes (cells/*μ*l)				
Mean ± SD	4905 ± 1951	4944 ± 1311	5649 ± 2102	
Median	4900	4900	5500	
Range	(1700–9100)	(2400–7000)	(1140–8200)	
Lymphocytes (%)				
Mean ± SD	19.1 ± 7.44	21.9 ± 5.2	30.4 ± 10.6	
Median	19.0	22.7	29.9	
Range	(8.0–33.0)	(14.9–33.5)	(18.1–47.2)	
Monocytes (%)				
Mean ± SD	9.3 ± 3.7	7.7 ± 2.6	6.8 ± 2.7	
Median	9.1	8.2	6.7	
Range	(4.4 – 15.0)	(3.0 – 13.1)	(1.0 – 10.3)	
Neutrophils (%)				
Mean ± SD	70.6 ± 8.3	68.1 ± 6.5	59.4 ± 10.7	
Median	72.6	67.6	62.5	**0.003**
Range	(53.0–82.7)	(53.6–8.8)	(43.5–73.3)	
Platelets (x10^3^cells/*μ*l)				
Mean ± SD	191.6 ± 83.1	204.9 ± 49.4	241.8 ± 34.9	
Median	205.0	223.5	249.0	
Range	(30–360)	(128–276)	(192–306)	
C3 (mg/dl)			–	
Mean ± SD	73.2 ± 31.8	82.3 ± 28.1		
Median	68.5	78.7		
Range	(29.4-130.4)	(28.8-132.4)		
C4 (mg/dl)			–	
Mean ± SD	16.4 ± 9.9	15.2 ± 5.9		
Median	13.5	14.3		
Range	(5.9-37.2)	(6.0-29.0)		
Anti-dsDNA (IU/ml)			–	
Mean ± SD	135.6 ± 188.3	234.4 ± 363.5		
Median	49.0	12.5		
Range	(12.3-570.0)	(7.7-1116.7)		
*Clinical variables*				
CLASI activity score			–	**0.01**
Mean ± SD	20.9 ± 8.2	13.4 ± 6.3		
Median	20.0	12.5		
Range	(9-37)	(2-29)		
*Treatment*				
Antimalarial (%)	14 (63.6)	13 (65)	–	
Prednisone (mg/day)	10	15	–	
Immunosuppressants (%)	13 (59)	12 (60)	–	

**Table 2:** Multiple linear regression models for patients with SCLE.

	Model	miRNAs	*R*	*R* square	*β*	*t*	Sig. (*P*)	95% IC for *β*	
								Lower	Up
Peripheral cells									
CD3^+/^CD4^+^/CD25^−^/IL-4^+^	1	miR-23b	0.493	0.244	-0.493	-2.407	0.027	-0.901	-0.061
CD3^+^/CD4^+^/CD25^+^/IFN-*γ*^+^	1	miR-1246	0.431	0.186	-0.431	-2.908	0.006	-0.399	-0.071
CD3^+^/CD4^+^/CD25hi/Foxp3^+^	1	miR-23b	0.449	0.201	-0.449	-2.13	0.047	-1.47	-0.01
Tissue cells									
IL-4-expressing CD4^+^ cells (epidermis)	1	miR-21	0.487	0.237	0.487	2.365	0.029	0.425	7.171
IFN-*γ*-expressing CD4^+^ cells (dermis)	1	miR-31	0.467	0.218	0.467	2.238	0.038	0.717	22.62
IL-10-expressing CD20^+^ cells (epidermis)	1	miR-21	0.492	0.242	0.492	2.396	0.028	0.61	9.298

SCLE: subacute cutaneous lupus erythematosus; DLE: discoid lupus erythematosus; HD: healthy donors.

**Table 3:** Multiple linear regression models for patients with DLE.

	Model	miRNAs	*R*	*R* square	*β*	*t*	Sig. (*P*)	95% IC for *β*	
								Lower	Up
Peripheral cells									
CD123hi/CD196^+^/IDO^+^	1	miR-150	0.448	0.201	0.448	2.125	0.048	0.009	1.54
Tissue cells									
IL-4-expressing CD4^+^ cells (dermis)	1	miR-150	0.517	0.267	0.517	2.562	0.020	0.27	2.728
IL-10-expressing CD20^+^ cells (epidermis)	1	miR-1246	0.75	0.563	0.75	4.811	<0.001	0.613	1.563
	2	miR-1246	0.809	0.655	0.859	5.672	<0.001	0.782	1.709
		miR-146a			-0.322	-2.13	0.048	-0.165	-0.001
IL-10-expressing CD20^+^ cells (epidermis)	1	miR-1246	0.736	0.542	0.736	4.611	<0.001	0.83	2.22
	2	miR-1246	0.673	0.634	0.866	5.874	<0.001	1.15	2.439
		miR-146a			-0.385	-2.612	0.018	-0.256	-0.027

SCLE: subacute cutaneous lupus erythematosus; DLE: discoid lupus erythematosus; HD: healthy donors.

## Data Availability

Data will be provided based on requirement.

## Additional Points

*What Is Already Known About This Topic*. (1) Cutaneous lupus erythematosus (CLE) is an autoimmune condition that comprehends a wide range of dermatological manifestations that can appear independently, or precede, systemic lupus erythematosus. (2) The pathogenesis of CLE is multifactorial and involves genetic predisposition, environmental factors, and abnormalities in the innate and adaptive immune response. (3) Recent studies have revealed the potential contribution of miRNAs in the regulation of signaling pathways. *What Does This Study Add*. (1) Circulating miR-150, miR-1246, and miR-21 were decreased in CLE patients. (2) SCLE variety was associated with lower levels of miR-21, miR-23b, and miR-31. (3) DLE variety was associated with lower levels of miR-146, miR-150, and miR-1246. These miRNAs drove the presence of different peripheral and skin subpopulations and participated in diverse pathways. *What Is the Translational Message*. (1) Circulating miRNAs could be used as future potential blood-based biomarkers to assess relapses and response to treatment, without the need of a skin biopsy. (2) Regulation of circulating miRNAs could be useful in the treatment of CLE.

## Supplementary Materials

Supplementary Materials Supplementary Figure 1: representative immunostaining of (a) IL-22-expressing cells, (b) IL-17A-expressing cells, (c) IL-4-expressing cells, (d) IFN-*γ*-expressing cells, (e) Foxp3-expressing cells, (f) IL-10-expessing cells, and (g) IDO-expressing cells in tissue biopsies from spleen. Original magnification was ×600. Supplementary Figure 2: heat map of KEGG pathways enriched with gene regulated by a combination of hsa-miR-150-5p, hsa-miR-1246, and hsa-miR-146a-5p in patients with DLE. Supplementary Figure 3: heat map of KEGG pathways enriched with gene regulated by a combination of hsa-miR-23b-3p, hsa-miR-21-5p, and hsa-miR-31-5p in patients with SCLE.
