# Supplementary material for: MicroRNA Expression in Cutaneous Lupus: A New Window to Understand Its Pathogenesis
Source: Mediators Inflamm. 2019 Dec 30;2019:5049245. doi: 10.1155/2019/5049245 (PMC7012207; doi:10.1155/2019/5049245)
Supplement: Supplementary Materials — Supplementary Figure 1: representative immunostaining of (a) IL-22-expressing cells, (b) IL-17A-expressing cells, (c) IL-4-expressing cells, (d) IFN-γ-expressing cells, (e) Foxp3-expressing cells, (f) IL-10-expessing cells, and (g) IDO-expressing cells in tissue biopsies from spleen. Original magnification was ×600. Supplementary Figure 2: heat map of KEGG pathways enriched with gene regulated by a combination of hsa-miR-150-5p, hsa-miR-1246, and hsa-miR-146a-5p in patients with DLE. Supplementary Figure 3: heat map of KEGG pathways enriched with gene regulated by a combination of hsa-miR-23b-3p, hsa-miR-21-5p, and hsa-miR-31-5p in patients with SCLE. [file 5049245.f1.pdf]

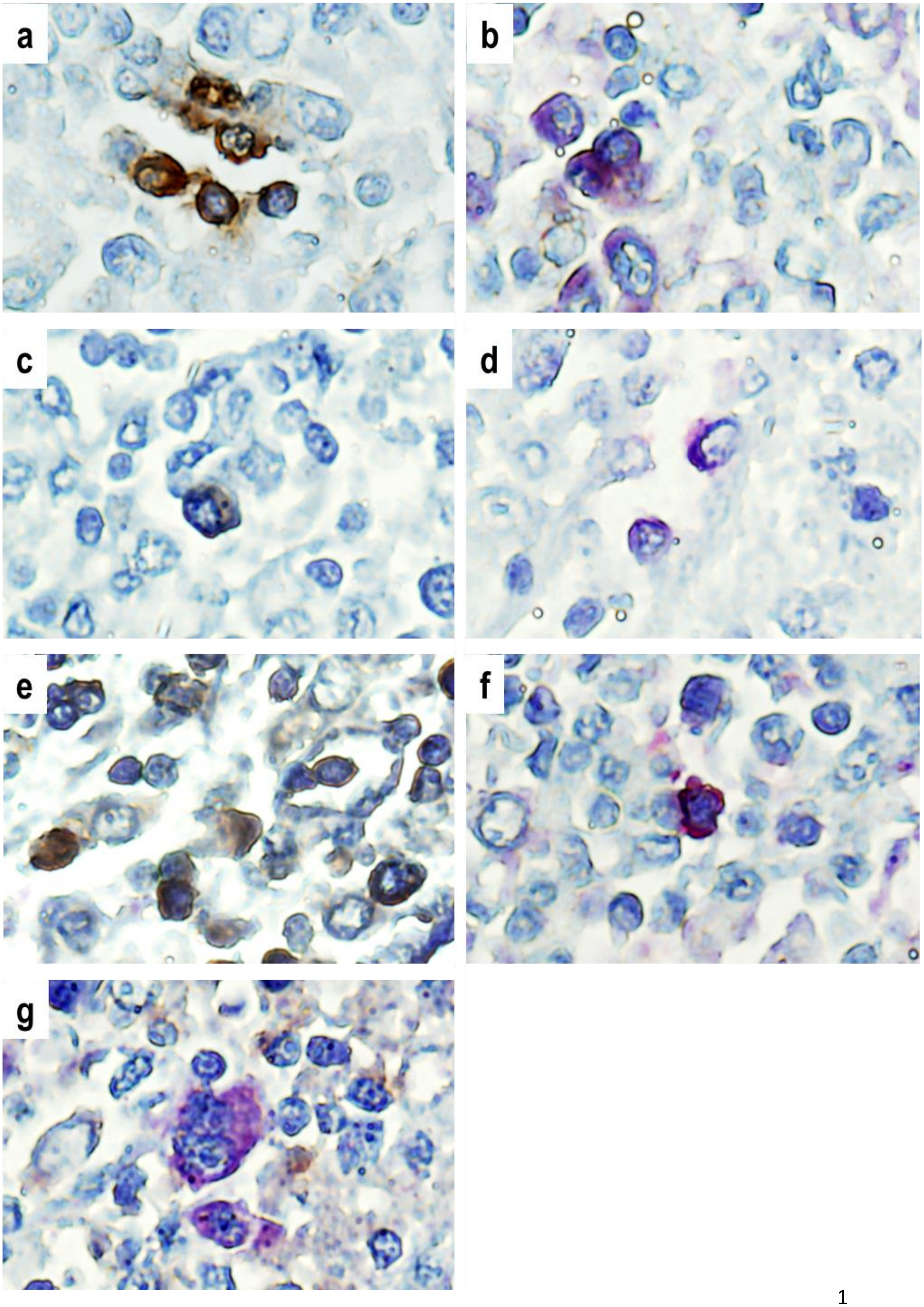
a
b
c
d
e
f
g

Supplementary Figure 1: Representative immunostaining of (a) IL-22-expressing cells, (b) IL-17A-expressing cells, (c) IL-4-expressing cells, (d) IFN-γ- expressing cells, (e) Foxp3-expressing cells, (f) IL-10-expessing cells and (g) IDO-expressing cells in tissue biopsies from spleen. Original magnification was X600.

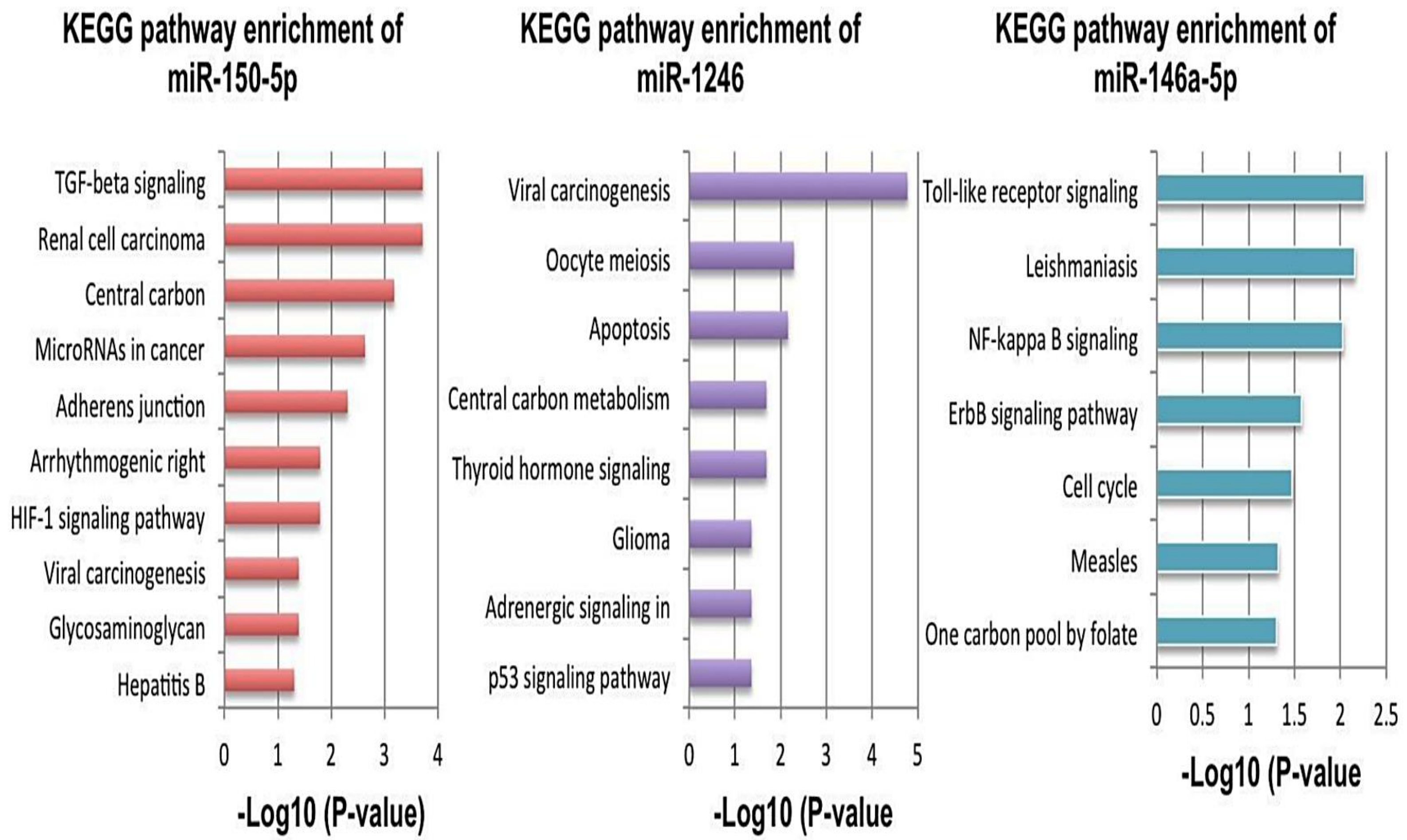
KEGG pathway enrichment of miR-150-5p
TGF-beta signaling
Renal cell carcinoma
Central carbon
MicroRNAs in cancer
Adherens junction
Arrhythmogenic right
HIF-1 signaling pathway
Viral carcinogenesis
Glycosaminoglycan
Hepatitis B
0 1 2 3 4
-Log10 (P-value)
KEGG pathway enrichment of miR-1246
Viral carcinogenesis
Oocyte meiosis
Apoptosis
Central carbon metabolism
Thyroid hormone signaling
Glioma
Adrenergic signaling in
p53 signaling pathway
0 1 2 3 4 5
-Log10 (P-value
KEGG pathway enrichment of miR-146a-5p
Toll-like receptor signaling
Leishmaniasis
NF-kappa B signaling
ErbB signaling pathway
Cell cycle
Measles
One carbon pool by folate
0 0.5 1 1.5 2 2.5
-Log10 (P-value

Supplementary Figure 2: Heatmap of KEGG pathways enriched with gene regulated by combination of hsa-miR-150-5p, hsa-miR-1246 and hsa-miR-146a-5p in patients with DLE.

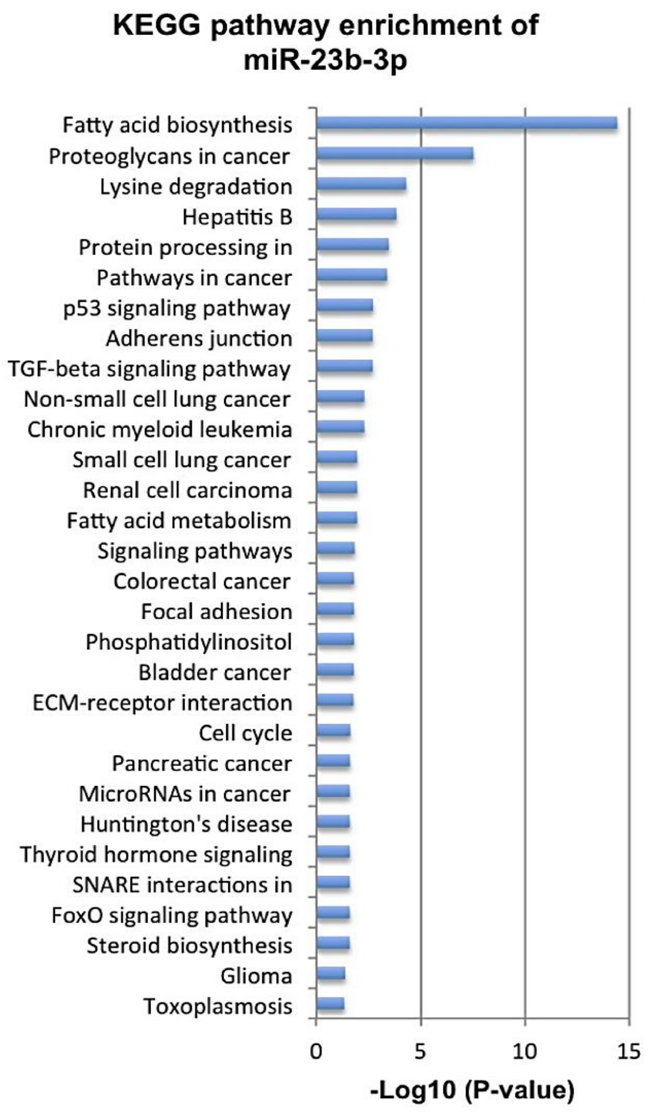
KEGG pathway enrichment of miR-23b-3p
Fatty acid biosynthesis
Proteoglycans in cancer
Lysine degradation
Hepatitis B
Protein processing in
Pathways in cancer
p53 signaling pathway
Adherens junction
TGF-beta signaling pathway
Non-small cell lung cancer
Chronic myeloid leukemia
Small cell lung cancer
Renal cell carcinoma
Fatty acid metabolism
Signaling pathways
Colorectal cancer
Focal adhesion
Phosphatidylinositol
Bladder cancer
ECM-receptor interaction
Cell cycle
Pancreatic cancer
MicroRNAs in cancer
Huntington's disease
Thyroid hormone signaling
SNARE interactions in
FoxO signaling pathway
Steroid biosynthesis
Glioma
Toxoplasmosis
0
5
10
15
-Log10 (P-value)

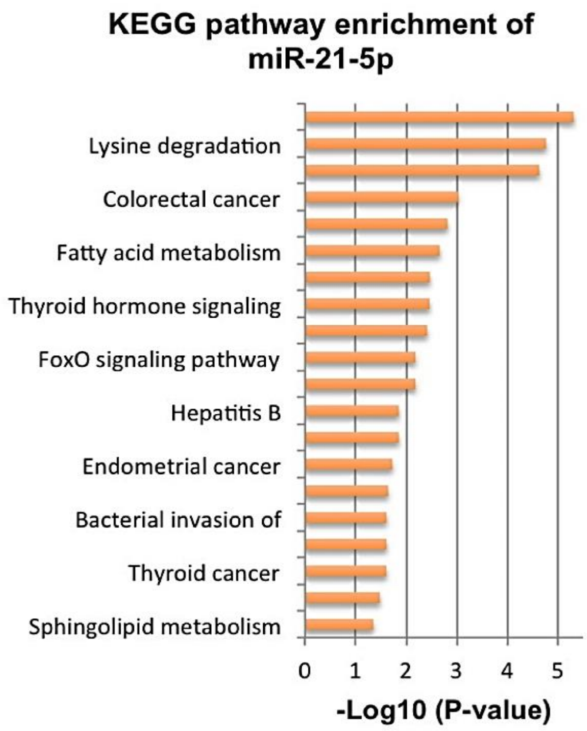
KEGG pathway enrichment of miR-21-5p
Lysine degradation
Colorectal cancer
Fatty acid metabolism
Thyroid hormone signaling
FoxO signaling pathway
Hepatitis B
Endometrial cancer
Bacterial invasion of
Thyroid cancer
Sphingolipid metabolism
0
1
2
3
4
5
-Log10 (P-value)

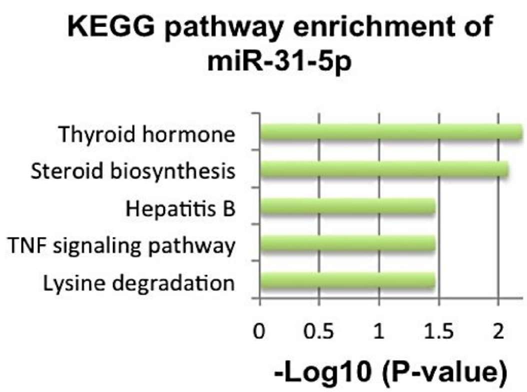
KEGG pathway enrichment of miR-31-5p
Thyroid hormone
Steroid biosynthesis
Hepatitis B
TNF signaling pathway
Lysine degradation
0
0.5
1
1.5
2
-Log10 (P-value)

Supplementary Figure 3: Heatmap of KEGG pathways enriched with gene regulated by combination of hsa-miR-23b-3p, hsa-miR-21-5p and hsa-miR-31-5p in patients with SCLE.
